# The Prediction of Elastic Modulus in Sheet-Reinforced Composites Using a Homogenization Approach

**DOI:** 10.3390/ma18081698

**Published:** 2025-04-08

**Authors:** Xiaoxia Zhai, Huzaifa Bin Hashim, Jun Huang, Eugene Zhen Xiang Soo

**Affiliations:** 1 Department of Civil Engineering, Faculty of Engineering, University of Malaya, Kuala Lumpur 50603, Malaysia; 17221873@siswa.um.edu.my; 2 College of Architecture and Civil Engineering, Nanning University, Nanning 530200, China; huangjun@unn.edu.cn; 3 Civil Engineering Programme, Faculty of Engineering, Universiti Malaysia Sabah, Kota Kinabalu 88400, Sabah, Malaysia; esoo@ums.edu.my

**Keywords:** Young’s modulus, sheet-reinforced composites, graphene composites, interface, representative volume element (RVE)

## Abstract

Based on the equal-stress formula and equal-strain formula of material mechanics, a new formula for predicting Young’s modulus of sheet-reinforced composite is derived by taking sheet-graphene composite as an example. The effectiveness of the formula is verified by comparing it with the mixing rate (ROM), Halpin–Tsai equation, and finite-element simulation. This formula is used to discuss the effect of interface-layer properties on the modulus of composite materials. Compared with the case without interface, when the graphene content is 3% and the interface-layer properties are linearly distributed and exponentially distributed, respectively, the embedded RVE modulus prediction increases by 5.06%, and the sandwich RVE modulus prediction increases by 56.5% and 31.75%, respectively. The influence of the change in interface-layer thickness from 0 to 1.5 nm (determined according to the existing literature) is also discussed. The predicted modulus of embedded RVE and sandwich RVE increases by 73% and 11.3%, respectively. The results show that the influence of the thickness and properties of the interface layer on the modulus prediction of graphene composites cannot be ignored. Combined with the analysis of experimental data, it is found that the experimental data fall within the prediction range of the modulus of the formula, indicating that the formula can be used for the preliminary trend analysis of the mechanical test of graphene-composite materials in the early stage, saving testing costs and time.

## 1. Introduction

Composites, formed by combining two or more materials with distinct properties, exhibit enhanced stiffness, strength, and thermal properties compared to their constituent materials [1]. For two-dimensional components such as plates and shells, sheet reinforcement offers superior efficiency compared to fiber reinforcement, as it provides support in all directions within the reinforcement plane [2]. Additionally, sheet materials exhibit higher stacking volume and lower expansion coefficients [3], making them economically and technically advantageous. Current sheet reinforcements include graphene and its derivatives, which have shown exceptional performance in composite materials [4,5,6,7,8,9]. This study focuses on graphene composites, conducting theoretical and numerical analyses of their elastic modulus.

The interface between the reinforcement and matrix plays a critical role in transferring deformation and load, significantly impacting the composite’s overall performance [10,11,12,13,14]. Due to the complex adhesive behavior induced by van der Waals forces at the interfaces of graphene composites, homogenization methods are commonly employed in theoretical analyses. While numerical simulations have been widely used to study the interface effect [12,15,16,17,18,19,20,21,22,23,24], theoretical formulas directly analyzing the interface layer are scarce. This paper addresses this gap by deriving a formula for predicting the Young’s modulus of graphene composites, considering the interface effect.

The numerical simulation is mainly used to construct a three-phase model [25,26,27] of the composite material, encompassing graphene, the matrix, and the interface layer. By utilizing the spring element to simulate the interfacial van der Waals interactions, Chandra et al. [28] developed a finite-element model to investigate the overall nonlinear behavior of composite materials and the failure mechanisms of polymer matrices; Chowdhury et al. [29] utilized a spring-based model to simulate the interaction between graphene and its surrounding matrix, and developed a hybrid finite-element three-phase model to investigate the vibration frequency of the nanoparticle composite. Montazeri and Rafii-Tabar et al. [30] employ a spring element to model the interaction between graphene sheets (GSs) and polymers, thereby establishing a framework to calculate the elastic constants of nanocomposites. Gates et al. [31] models the nanotube/polymer interface as an effective continuum fiber and develops a constitutive model for single-walled carbon nanotube (SWNT)-reinforced polymer composites. This model predicts the overall mechanical properties of SWNT/polymer composites, considering variations in nanotube length, concentration, and orientation. The representative volume element (RVE) of the SWCNT-PVC system was modeled as a tri-phase composite by Spanos and Georgantzinos et al. [21], thereby validating that the interfacial properties of the non-crystalline matrix in carbon-nanotube composites can be approximated as a function of density. Zhangxin Guo et al. [32] uses C3D8 elements to model the interface region as a continuous material with specified thickness, enabling a 3D multi-scale simulation to study the mechanical properties of graphene-reinforced polymer-matrix composites. These studies demonstrate that incorporating the interface action is crucial for investigating the effective properties of composite materials. Numerical simulations of the interface have produced reliable results, but theoretical formulas for directly analyzing the interface layer are scarce.

The representative volume element (RVE) method [1] is extensively utilized to investigate the effective properties of nanocomposites [17,18,22,24,28,30,31,32,33,34,35,36,37,38]. Currently, the most commonly utilized representative volume element types primarily include the sandwich model [33,34,39,40,41,42] and the embedded model [33,34,36,41]. In the sandwich model, the graphene sheet and matrix are regarded as laminated composites, and the corresponding analytical formula is the rule of mixture (ROM) [28,33,34,40,41,42,43,44]; the embedded model refers to a configuration where the graphene sheet is fully encapsulated within the matrix, and its mechanical properties are estimated [45,46,47] using the Halpin–Tsai formula [48,49,50,51]. Both the ROM formula [1] and the Halpin–Tsai formula [49] have demonstrated significant utility in the field of composite materials mechanics, yielding reliable and consistent results [50,51,52]. Liu and Chen [33,34] investigated carbon-nanotube composites using both the sandwich model and the embedded model. The findings revealed that, for identical graphene size and content, the computational outcomes of these two models exhibited significant discrepancies. In the context of composite material research, the Halpin–Tsai empirical formula exhibits variations in its coefficients and values depending on diverse experimental conditions [49,50,52]. For the same composite material, the selection of representative volume elements can be progressively transitioned from a sandwich model to an embedded model, yet distinct equations must be employed for accurate numerical computation.

According to the structural characteristics of sheet graphene, a material mechanics formula for calculating the elastic modulus of composite materials is proposed based on the assumption of homogeneity under the premise that the matrix is stable at room temperature. The validity of the formula is verified by comparison with the ROM formula, Halpin–Tsai formula, and finite-element simulation results. This formula not only incorporates the interface interaction directly but also captures the continuous variation in the elastic modulus of the representative volume element as it transitions from an embedded model to a sandwich model. Additionally, the influence of interface properties and the thickness of the interface layer on the overall modulus of the composites is examined. By analyzing existing experimental data from the literature, it is demonstrated that the formula can define the range of modulus values and facilitate preliminary trend analysis for the mechanical testing of sheet-reinforced composite materials, thereby reducing experimental costs and time.

## 2. The Fundamental Theory

### 2.1. Mechanical Theory of Materials

**Figure 1:**
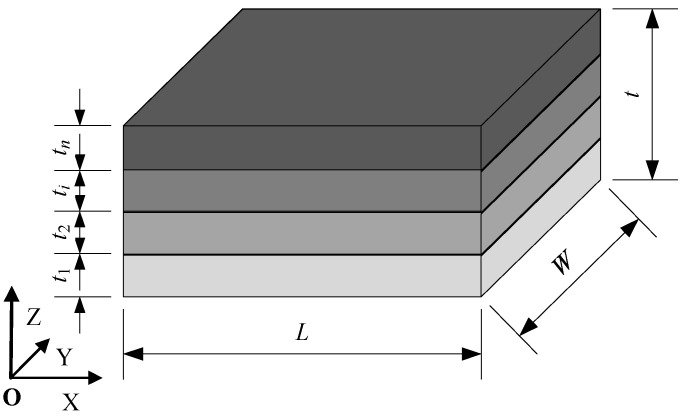
Composite materials.

The composite material, consisting of n different materials as depicted in Figure 1, has a length of L and a width of W. Each layer has thicknesses of $t_{1},t_{2},\cdots ,t_{n}$, respectively, resulting in a total model thickness of t. It is assumed that all layers of materials are isotropic and homogeneous materials with a Young’s modulus of $E_{1},E_{2},\cdots ,E_{n}$, respectively. When subjected to uniaxial tensile loading, the entire research object can be considered as experiencing equal-stress or equal-strain conditions. Therefore, the research object can be divided into an equal-stress model and an equal-strain model.

#### 2.1.1. Equal-Stress Model

**Figure 2:**
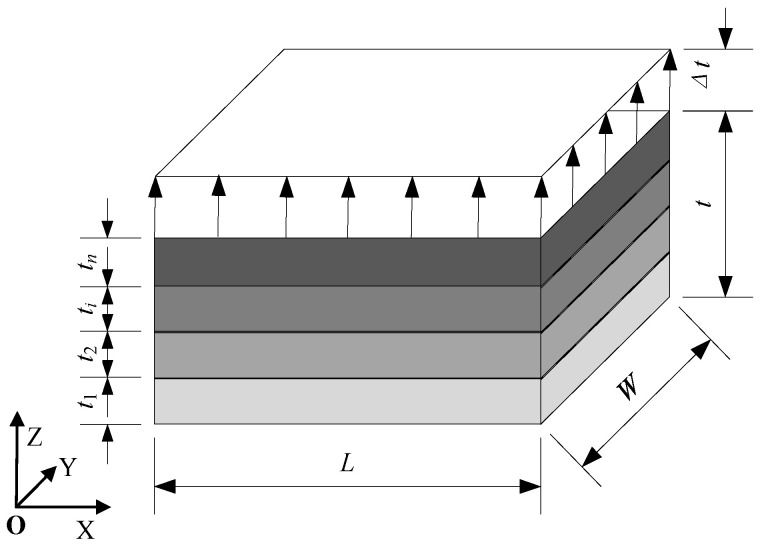
Equal-stress model.

The equal-stress model is depicted in Figure 2. The material undergoes stretching along the Z direction, with the Z = 0 plane being fixed, and its displacement constrained in the Z direction. Moreover, a small displacement of Δt is applied to the Z = t plane. To eliminate any rigid body displacement, both the X = 0 plane’s X direction displacement and the Y = 0 plane’s Y direction displacement is constrained. Assuming a uniform internal stress of σ in each layer of the material, the equivalent Young’s modulus in the Z direction for the laminated material can be determined as follows:

(1) $$E_{cz}=\frac{\sigma }{\bar{\varepsilon }}$$

where $\bar{\varepsilon }$ is the equivalent strain in the Z direction of the laminated material, which can be calculated by the following formula:

(2) $$\bar{\varepsilon }=\frac{\sum_{i=1}^{n}\Delta t_{i}}{\sum_{i=1}^{n}t_{i}}=\frac{1}{t}\sum_{i=1}^{n}t_{i}\varepsilon_{i}$$

(3) $$\varepsilon_{i}=\frac{\sigma }{E_{i}}$$

where t is the total thickness of the laminated material; $t_{i}$ is the thickness of the i layer material; $\Delta t_{i}$ is the thickness change in the i layer material; and $\Delta t_{i}$ is the strain of the i layer material. Substituting Formulas (3) and (2) into Formula (1) gives:

(4) $$E_{cz}=\frac{1}{\sum_{i=1}^{n}\frac{V_{i}^{fr}}{E_{i}}}$$

(5) $$V_{i}^{fr}=\frac{t_{i}}{t}$$

where $V_{i}^{fr}$ represents the volume content of the i layer material.

Formula (5) can be transformed into the following expression when substituting Formula (4) into it, given that only two materials are included.

(6) $$E_{cx}=\frac{E_{1}E_{2}}{E_{2}V_{1}^{fr}+E_{1}V_{2}^{fr}}$$

#### 2.1.2. Equal-Strain Model

**Figure 3:**
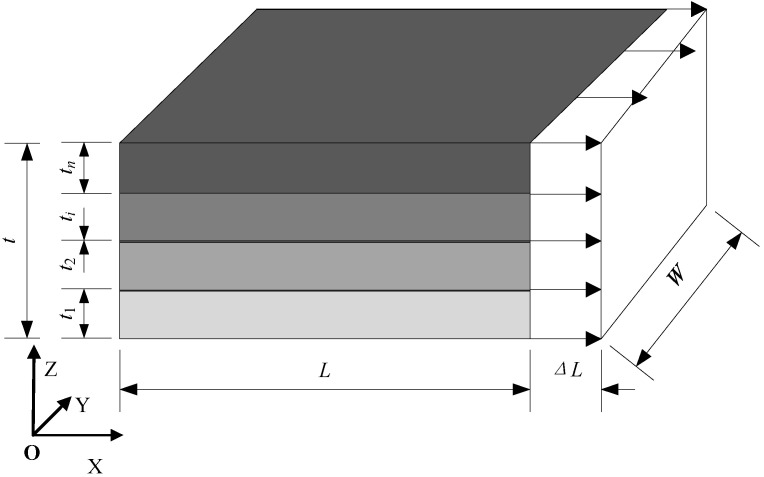
Equal-strain model for laminated materials.

The equal-strain model is illustrated in Figure 3. Uniaxial stretching is applied to the composite material along the X direction, where the displacement of the X = 0 plane in the X direction is constrained and a small displacement (denoted as ΔL) is imposed on the X = L plane. To eliminate any rigid body displacement, both the Y = 0 plane’s Y direction displacement and the Z = 0 plane’s Z direction displacement is constrained. According to the given boundary conditions, the global strain ε is in accordance with the strain $\varepsilon_{i}$ of each layer; that is, $\varepsilon =\varepsilon_{i}$. Consequently, the equivalent Young’s modulus $E_{cx}$ in the X direction of the composite material can be determined as follows:

(7) $$E_{cx}=\frac{\bar{\sigma }}{\varepsilon }$$

where $\bar{\sigma }$ is the equivalent stress on the X section, which can be determined using the following formula.

(8) $$\bar{\sigma }=\frac{\sum RF_{i}}{W\sum t_{i}}=\frac{\sum E_{i}t_{i}\varepsilon }{\sum t_{i}}$$

(9) $$\varepsilon =\frac{\Delta L}{L}$$

where $RF_{i}$ is the support reaction force of the i layer material during stretching. By substituting Equations (8) and (9) into Equation (7), we obtain:

(10) $$E_{cx}=\sum E_{i}V_{i}^{fr}$$

In the formula, $V_{i}^{fr}$ represents the volume content of the material in the i layer, which is expressed as follows:

(11) $$V_{i}^{fr}=\frac{t_{i}}{t}$$

If there are only two materials, the Young’s modulus is $E_{1}$ and $E_{2}$, respectively, which can be sorted by Equation (10):

(12) $$E_{cx}=E_{1}V_{1}^{fr}+E_{2}V_{2}^{fr}$$

It can be seen that Formula (12) is the rule of mixture (ROM).

### 2.2. Derivation of the Mixed Formula

**Figure 4:**
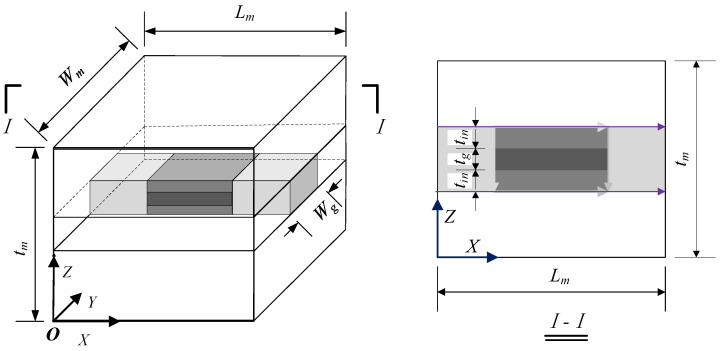
Three-phase embedded RVE.

When considering the interface effect, the RVE model of the graphene composite is illustrated in Figure 4. Under the assumption of equal strain, the interface layer and graphene are considered in parallel. Subsequently, under the assumption of equal stress, the regions adjacent to the graphene on both sides, with equivalent thickness to the matrix, are treated as a series connection. Finally, this series connection is paralleled with the remaining matrix. Based on the equal-strain assumption, the formula for calculating the three-phase model of Young’s modulus for the graphene composite is derived.

In Figure 4, the displacement in the X direction of the X = 0 plane is constrained, while a tiny displacement ΔL is applied to the X = L plane. Considering the parallel association between the interface layer and graphene, the elastic modulus in the X direction can be expressed as $E_{cx}^{1}$, using the equal-strain model formula as follows:

(13) $$E_{cx}^{1}=E_{g}V_{g}^{fr}+E_{in}V_{in}^{fr}$$

wherein $V_{g}^{fr}$ represents the volume fraction of graphene, $E_{g}$ denotes the elastic modulus of the graphene sheet, $V_{in}^{fr}$ is the volume fraction of the interfacial phase in a union, and $E_{in}$ is the elastic modulus of interfacial phase. There:

(14) $$V_{g}^{fr}=\frac{L_{g}W_{g}t_{g}}{L_{g}W_{g}\times (t_{g}+2t_{in})}$$

(15) $$V_{in}^{fr}=1-V_{g}^{fr}$$

where $L_{g}$, $W_{g}$, and $t_{g}$ are the length, width, and thickness of the graphene sheet, respectively, and $L_{m}$, $W_{m}$, and $t_{m}$ are the length, width, and thickness of the matrix, respectively. By substituting Formulas (14) and (15) into Formula (13), we obtain:

(16) $$E_{cx}^{1}=\frac{E_{g}t_{g}+2E_{in}t_{in}}{t_{g}+2t_{in}}$$

Based on the assumption of equal stress, a series body is formed by combining the parallel matrix with the matrix of equal thickness, and the elastic modulus in the X direction is expressed as $E_{cx}^{2}$. The formula of the equal-stress model is as follows:

(17) $$E_{cx}^{2}=\frac{E_{cx}^{1}E_{m}}{E_{m}V_{1}^{fr}+E_{cx}^{1}V_{2}^{fr}}$$

where $V_{1}^{fr}$ is the volume fraction of the combined body in a series, $V_{2}^{fr}$ is the volume fraction of the remaining matrix in the parallel body, and $E_{m}$ indicates the elastic modulus of the matrix. Among these variables:

(18) $$V_{1}^{fr}=\frac{L_{g}}{L_{m}}$$

(19) $$V_{2}^{fr}=1-V_{2}^{fr}$$

By substituting Equations (18) and (16) into Equation (17), the following is obtained:

(20) $$E_{cx}^{2}=\frac{\left[\frac{E_{g}t_{g}+2E_{in}t_{in}}{\left(t_{g}+2t_{in}\right)}\right]E_{m}L_{m}}{E_{m}L_{g}+\left[\frac{E_{g}t_{g}+2E_{in}t_{in}}{\left(t_{g}+2t_{in}\right)}\right]L_{m}-\left[\frac{E_{g}t_{g}+2E_{in}t_{in}}{\left(t_{g}+2t_{in}\right)}\right]L_{g}}$$

Finally, based on the equal-strain model, the series and the surrounding matrix are connected in parallel. By substituting Equation (20) into Equation (12), the elastic modulus of the whole RVE is denoted as Ecx and calculated as:

(21) $$E_{cx}=E_{cx}^{2}V_{2^{\prime }}^{fr}+E_{m}(1-V_{2^{\prime }}^{fr})$$

where $V_{2^{\prime }}^{fr}$ is the volume fraction of the series in RVE, which is calculated as follows:

(22) $$V_{2^{\prime }}^{fr}=\frac{W_{g}\times (t_{g}+2t_{in})}{W_{m}t_{m}}$$

With the substitution of Equations (22) and (20) into Equation (21), the formula for calculating the elastic modulus in the X direction of the three-phase model can be summarized as follows:

(23) $$\begin{array}{l} E_{cx}=\frac{\left(E_{g}E_{m}t_{g}L_{m}W_{g}+E_{in}E_{m}2t_{in}L_{m}W_{g}\right)}{E_{m}L_{g}W_{m}t_{m}+\left[\frac{E_{g}t_{g}+2E_{in}t_{in}}{\left(t_{g}+2t_{in}\right)}\right]L_{m}W_{m}t_{m}-\left[\frac{E_{g}t_{g}+2E_{in}t_{in}}{\left(t_{g}+2t_{in}\right)}\right]L_{g}W_{m}t_{m}}\\ \;+E_{m}\times (1-\frac{W_{g}\times (t_{g}+2t_{in})}{W_{m}t_{m}}) \end{array}$$

### 2.3. Degradation of the Mixed Formula

#### 2.3.1. The Three-Phase Model Degenerates into a Two-Phase Model

**Figure 5:**
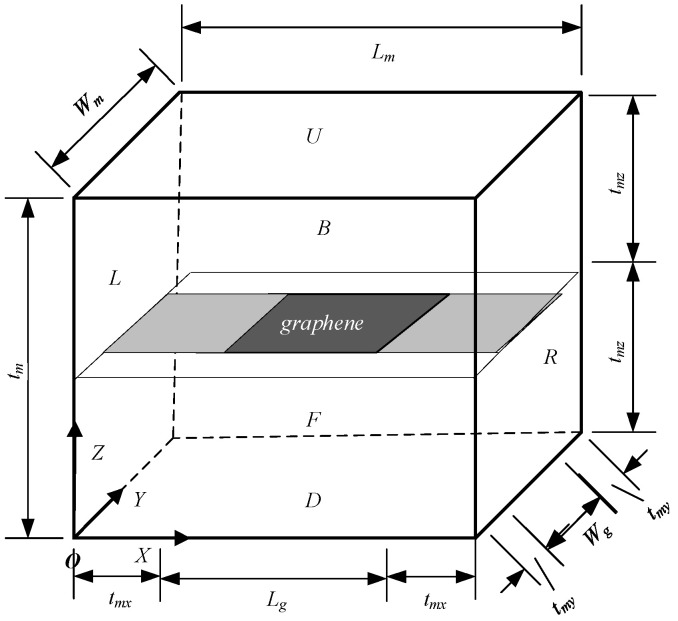
Two-phase embedded RVE.

If the interface effect is ignored, that is, $t_{in}=0,E_{in}=0$, then the graphene composite is shown in Figure 5, which is a typical two-phase model. By sorting out Formula (23), we obtain:

(24) $$E_{cx}=\frac{E_{g}E_{m}t_{g}L_{m}W_{g}}{W_{m}t_{m}\left(E_{m}L_{g}-E_{g}L_{g}+E_{g}L_{m}\right)}+E_{m}\times (1-\frac{W_{g}t_{g}}{W_{m}t_{m}})$$

This represents the mixing formula for the two-phase model of composite materials, demonstrating that the three-phase mixing formula can be simplified to the two-phase mixing formula.

#### 2.3.2. The Mixed Formula Degenerates into ROM

Based on the two-phase model formula obtained in Section 2.3.1, it is further degraded into ROM. When $L_{m}=L_{g},W_{m}=W_{g}$, that is, the laminated material, the value is substituted into the Formula (24) to sort out:

(25) $$E_{cx}=E_{g}\times \frac{t_{g}}{t_{m}}+E_{m}\times (1-\frac{t_{g}}{t_{m}})$$

For laminated materials, $V_{g}^{fr}=\frac{t_{g}}{t_{m}}$ is the volume fraction of the graphene reinforcement. Formula (25) can be written as:

(26) $$E_{cx}=E_{g}\times V_{g}^{fr}+E_{m}\times (1-V_{g}^{fr})$$

Now, the mixed formula is consistent with the rule of mixture (ROM) [1].

## 3. Finite-Element Simulation of Graphene Composites

### 3.1. Calculation Model and Parameters

**Figure 6:**
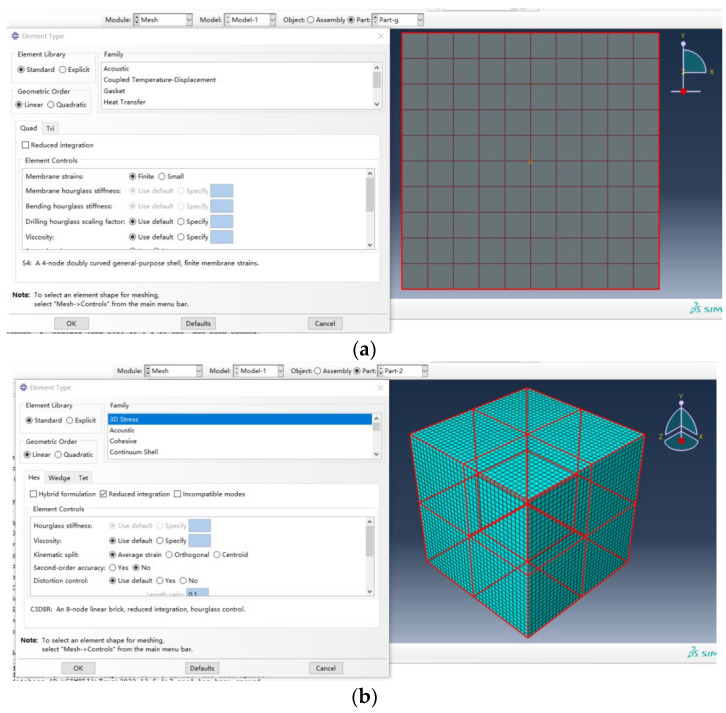

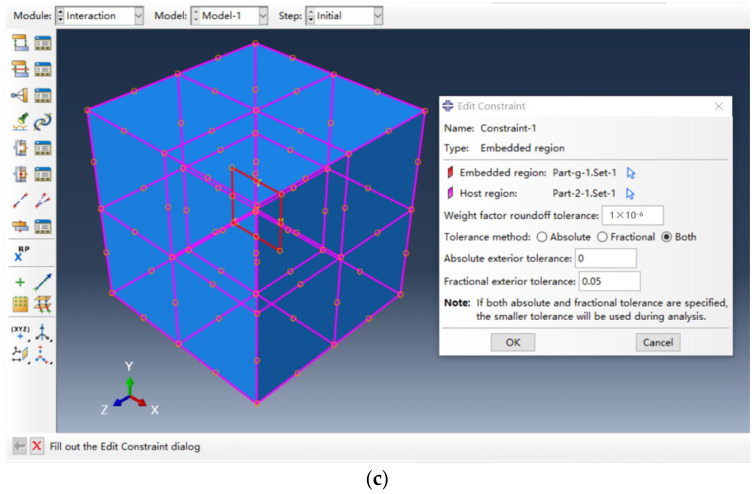
The two−phase model of graphene polymer: (**a**) the meshing of the reinforcement and cell selection; (**b**) the meshing of the matrix and cell selection (**c**) the bonding of the two phases.

The finite-element modeling analysis was conducted using ABAQUS 6.14 software. Specifically, the shell element (S4) was employed to simulate the graphene, while the 8-node solid element (C3D8) was utilized to model the matrix material, as illustrated in Figure 6. In the grid division, for the convenience of calculation, the size is selected as 0.1 times the size of the reinforcement, and the grid size of the matrix is the same as that of the reinforcement. Assuming that the connection between graphene and polymer is smooth, the embedded regional contact mode of software is adopted, and the matrix is set as the main body and the graphene reinforcement is set as the embedded object, and the two-phase model of graphene composite is established.

### 3.2. Boundary-Condition Setting

**Figure 7:**
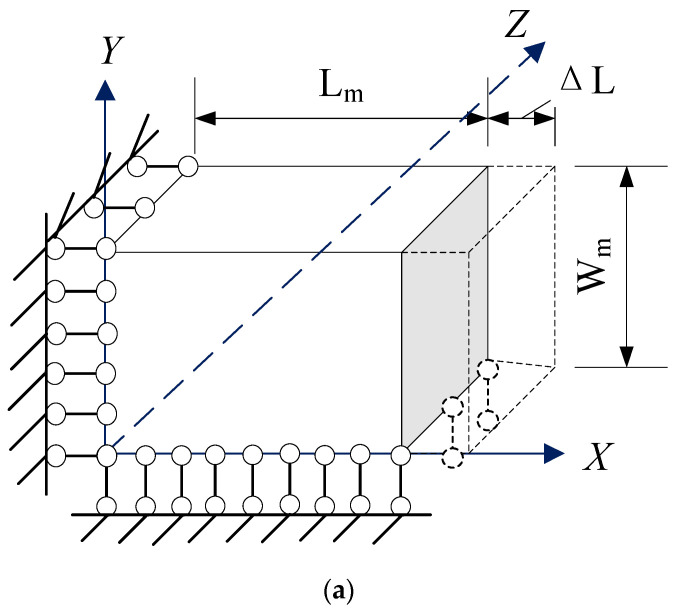

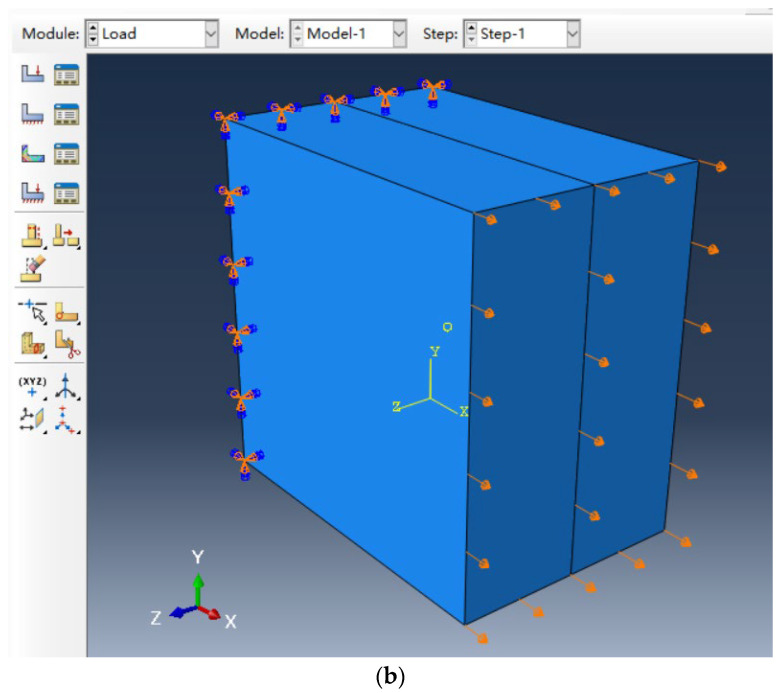
Boundary conditions of RVE. (**a**) Theoretical boundary condition. (**b**) Model boundary condition.

As illustrated in Figure 7, the boundary conditions impose constraints on the displacement in the X direction on the X=0 plane. A small displacement ΔL is applied on the $X=L_{m}$ plane, while the Y-direction displacement is restricted on the Y=0 plane. All other surfaces remain unconstrained.

## 4. Results and Discussion

### 4.1. Comparative Analysis of Young’s Modulus Prediction

#### 4.1.1. Predictive Comparison of Analytical Solutions

The rule of mixture (ROM) and Halpin–Tsai method have been widely used for composites’ elastic properties prediction in many studies. ROM is applicable to laminated materials and the Halpin–Tsai method is applicable to composites with embedded reinforcements, and their expressions are as followed:

The rule of mixture (ROM) expression is:

(27) $$E_{cx}=E_{1}V_{fr1}+E_{2}V_{fr2}$$

where $E_{cx}$ is the elastic modulus of composite materials; $E_{1}$, $E_{2}$ are the elastic modulus of the first and second phase materials, respectively; $V_{fr1}$, $V_{fr2}$ are volume fractions of the first and second phase materials, respectively.

The Halpin–Tsai formula expression [52] is:

(28) $$\begin{array}{l} E_{cx}=\left[\frac{1+\frac{2}{3}\zeta_{1}\eta_{L}V_{fr}^{g}}{1-\eta_{L}V_{fr}^{g}}\right]E_{m}\\ \eta_{L}=\frac{\zeta_{2}-1}{\zeta_{2}+\frac{2}{3}\zeta_{1}}\\ \zeta_{1}=\frac{L_{g}}{t_{g}}\\ \zeta_{2}=\frac{E_{g}}{E_{m}} \end{array}$$

The symbols in the formula express the same meaning as before.

In this section, the Youngs’ modulus *E*_cx_ of graphene nanocomposite predicted by the presented method are compared with those calculated by ROM and the Halpin–Tsai method, and three sizes (*L*_g_ = *W*_g_ = 1 nm, 5 nm, 10 nm) and three volume contents (*V*_g_ = 1%, 5%, 10%) of graphene are discussed, as shown in Table 1 and Table 2, respectively.

The proposed formula’s predictions were compared with the rule of mixture (ROM) and Halpin–Tsai methods. For graphene nanocomposites with varying sizes (Lg = Wg = 1 nm, 5 nm, 10 nm) and volume fractions (Vg = 1%, 5%, 10%), the results showed excellent agreement with ROM and Halpin–Tsai predictions, with a maximum deviation of only 3.3% (Table 1 and Table 2).

According to Section 2.3.2, when Lm = Lg, Wm = Wg, the new formula is completely consistent with the ROM formula, so for the sandwich model in Table 1, the calculation results of the mixed formula are completely consistent with the ROM calculation results. The H–T formula [52] chosen in this example is suitable for predicting the modulus of short-fiber or particle-reinforced composite materials. For this kind of discontinuous reinforced composite material, the representative volume unit selected in this paper is the embedded RVE that completely wraps the matrix around the reinforcement body. By selecting appropriate RVE dimension parameters, the prediction results of this formula are close to the H–T calculation results. The validity of the solution of this formula is verified.

#### 4.1.2. Comparison with FEM

**Figure 8:**
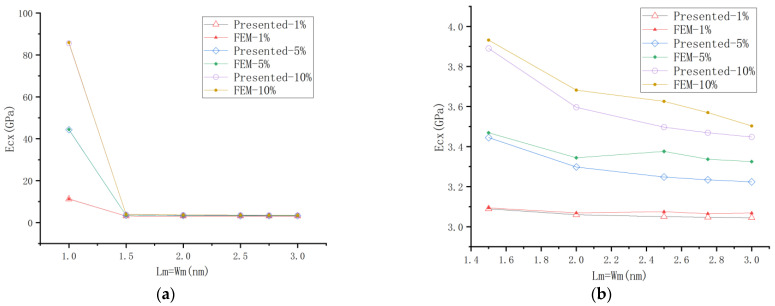
Young’s modulus predicted by the presented method and FEM. (**a**) From sandwich models to embedded models. (**b**) Data comparison of embedded models.

The FEM is a commonly used numerical simulation method for predicting the elastic mechanic properties of graphene nanocomposites. The Young’s modulus predicted by FEM are shown in Figure 8, and is compared with the presented method. The graphene composite with a size of Lg = Wg = 1 nm, and graphene contents *V*_g_ = 1%, 5%, 10% are discussed. As seen in Figure 8a, the presented results have good agreement with the FEM results.

### 4.2. Interface Effect

#### 4.2.1. Influence of Interface on Prediction Results of Elastic Modulus

The influence of the interface layer is discussed in this example. In general, the graphene interface is considered a singular material interphase, with the interphase layer generally characterized by either linear [24] or exponential [20] distribution models.

The representation of the linear distribution interface [24] is as follows:

(29) $$E_{in}\left(z\right)=E_{g}+\left(E_{m}-E_{g}\right)\frac{z}{t_{in}}\begin{array}{cc} & \left(-t_{in}\leq z\leq t_{in},z\neq 0\right) \end{array}$$

where z represents the direction of gradient change in material properties, also known as thickness direction; E represents the Young’s modulus of the material. The subscripts in, m, and g represent the interface layer, matrix, and graphene, respectively, where z = 0 is the position of the graphene midplane and t_in_ is the thickness of the interface layer.

The expression of the exponential distribution interface [20] is:

(30) $$E_{in}\left(z\right)=Aze^{-Cz}+B$$

where A, B, and C represent the coefficients associated with the properties of graphene and the matrix material. It is assumed that there is a continuous gradation of material properties in the thickness direction, transitioning from those of graphene to those of the matrix material. Based on this assumption, the subsequent boundary conditions can be derived:

(31) $$E_{in}\left(+t_{in}\right)=E_{in}\left(-t_{in}\right)=E_{m}$$

(32) $$E_{in}\left(0^{+}\right)=E_{in}\left(0^{-}\right)=E_{g}$$

(33) $${\left.\frac{dE_{in}\left(z\right)}{dz}\right|}_{z=+t_{in}}={\left.\frac{dE_{in}\left(z\right)}{dz}\right|}_{z=-t_{in}}=0$$

The coefficients A, B, and C are obtained by solving the equations of the above three equations:

(34) $$A=\frac{e}{t_{in}}\left(E_{m}-E_{g}\right)$$

(35) $$B=E_{g}$$

(36) $$C=\frac{1}{t_{in}}$$

Substituting the values of A, B, and C into Formula (39), we obtain:

(37) $$E_{in}\left(z\right)=\frac{z}{t_{in}}e^{1-z/t_{in}}\left(E_{m}-E_{g}\right)+E_{g}\begin{array}{cc} & \left(-t_{in}\leq z\leq t_{in},z\neq 0\right) \end{array}$$

The thickness of the graphene interphase was 0.17 nm [20,22,24], with a graphene reinforcement size of Lg = Wg = 1 nm. The modulus of the graphene reinforcement was Eg = 829 GPa [43], while the matrix modulus was Em = 3 GPa [20]. To investigate the elastic modulus of composite materials under three different interface properties, both sandwich RVE and embedded RVE models were utilized. The embedded representative volume element is cuboidal in shape, with dimensions provided in Table 3. The three interface properties considered are non-interfacial phase, the linear distribution of interface attributes, and the exponential distribution of interface attributes, as detailed in Table 4 and Table 5. 

The predicted modulus values of the sandwich representative volume-unit model are presented in Table 4. It is evident that the properties of the interface layer significantly influence the elastic modulus of the composite material. Specifically, when the volume fraction of graphene is only 1%, the predicted results considering a linear interface are 46.45% higher than those without interfaces, while the predicted results with an exponential interface are 26.11% higher. This indicates that the interface-layer properties of laminated composites have a substantial impact on their elastic modulus and cannot be overlooked. The data clearly demonstrate that accounting for interface properties markedly affects the prediction of the elastic modulus of laminates.

The predicted modulus values of the embedded representative volume-unit model are presented in Table 5. It is evident that the influence of interface-layer properties on the elastic modulus of the composite material intensifies with an increase in graphene volume content. Notably, both linear and exponential interfaces exert a similar effect. When Vg equals 1%, the predicted modulus of the interface-inclusive model is 1.64% higher than that of the model without an interface, resulting in a deviation of 9.34%. This indicates that the impact of interface properties on the elastic modulus of sheet-reinforced composites is significant and cannot be overlooked. Therefore, considering interface properties is crucial for accurately predicting the elastic modulus of such composites.

#### 4.2.2. Effect of Interface Thickness on Elastic Modulus

Currently, the interface-layer thickness commonly employed in various studies is 0.17 nm [20,22,24], 0.265 nm [21], 0.28 nm [53], 0.34 nm [32], and 1.3 nm [54].

**Figure 9:**
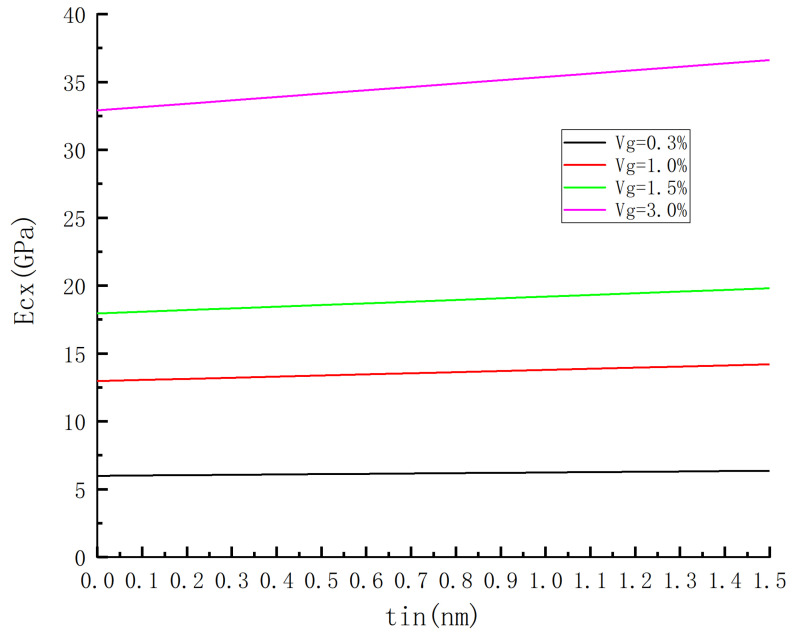
The influence of interface thickness on the predicted elastic modulus of sandwich model under different volume fractions.

**Figure 10:**
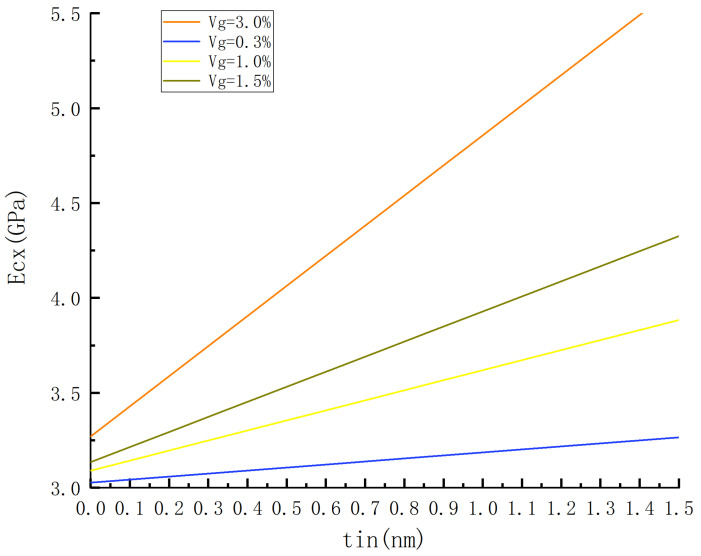
The influence of interface thickness on the predicted elastic modulus of embedded model under different volume fractions.

In this particular example, the mixing formula is utilized to investigate the impact of interface thickness on the elastic modulus of composite materials under volume fractions of 0.3 vol%, 1.0 vol%, 1.5 vol%, and 3 vol%. The corresponding results are presented in Figure 9 and Figure 10.

For instance, a sheet graphene with dimensions of 10 × 10 (nm) was employed with an elastic modulus of 1000 GPa, a matrix modulus of 3 GPa, and an interfacial layer modulus of 17 GPa [21]. Two different models were adopted for RVE: a sandwich model with dimensions of 10 × 10 (nm) and an embedded model with dimensions 15 × 15 (nm).

The increase in interface thickness is observed to result in an increase in Ecx, as depicted in Figure 9 and Figure 10. This can be attributed to the assumption that the interface modulus exceeds the matrix modulus, leading to an enhanced strengthening effect and elastic modulus with increasing interface thickness. Moreover, it is evident that the slope of the modulus curves for different graphene contents increases as the interfacial-layer tin thickens, with a further rise corresponding to higher graphene content. These findings highlight the significant influence of interfacial-layer thickness on Ecx, particularly when accompanied by greater graphene content.

By comparing Figure 9 and Figure 10, it is observed that the slope of the curve in Figure 9 is greater than that in Figure 9 for the same content. From Figure 10, It can be seen from Figure 10 that when tin increases from 0 to 1.5 nm with a volume fraction of 3%, the RVE model changes from a two-phase model to a three-phase model, and Ecx increases by 73% from 3.27 GPa to 5.65 GPa. Similarly, when tin increases within the same volume fraction range (3%) from zero to 1.5 nm, the RVE model transforms from a two-phase structure into a three-phase structure, leading to an increase in Ecx from 32.91 GPa to 36.62 GPa, representing an increment of 11.3%. Furthermore, it should be noted that interface thickness has a more pronounced impact on embedded models compared to sandwich models.

The influence of the interface layer on the composite’s elastic modulus was investigated. For sandwich models, the predicted modulus considering a linear interface was 46.45% higher than that without an interface, while the exponential interface resulted in a 26.11% increase (Table 4). For embedded models, the interface effect became more pronounced with increasing graphene content, with a maximum deviation of 9.34% (Table 5). These findings highlight the significant impact of interface properties on the composite’s modulus.

### 4.3. The Application of the Mixed Formula

#### 4.3.1. Determine the Range of Solutions of the Mixed Formula

It is evident from Figure 8a of Example 4.1.2 that the selection of the size of the representative volume element (RVE) significantly influences the predicted elastic modulus of the composite material. When the sandwich model is adopted, Ecx attains its maximum value. Under identical volume concentrations, as Lm increases, the Ecx value initially exhibits a sharp decline followed by a gradual stabilization. Given that the matrix thickness cannot be less than the reinforcement thickness, the side length of the graphene RVE cannot approach infinity. Consequently, when the RVE thickness reaches its minimum value of tm = 0.34 nm, data convergence across all experimental groups, as shown in Table 6, indicates that the composite’s elastic modulus ultimately converges to a value exceeding that of the matrix modulus.

It is evident from the data presented in Table 6 that for varying reinforcement sizes, under different volume fractions of each RVE, the minimum predicted elastic modulus converges to a value that exceeds the matrix modulus. This converged value is solely dependent on the volume fraction of the reinforcement. The enhancement in this model is achieved through edge force transfer rather than in-plane force transfer, which aligns with the fundamental principles of the model.

#### 4.3.2. Comparison with Experimental Data

Considering the interface effect, the results of predicting the elastic modulus of composites by using the mixed formula are compared with the existing literature and experiments. The interface properties were set as follows: a thickness of 0.265 nm, a density of 1.84 g·cm^−3^, a Young’s modulus of 17 GPa, and a Poisson’s ratio of 0.4 [21].

Eleven groups of experimental data were found from the existing literature, as shown in Table 7.

**Figure 11:**
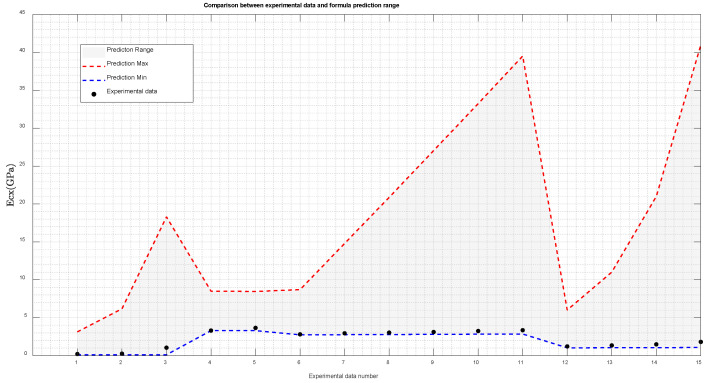
Comparison between the presented results and the experimental data.

Based on the conclusions presented in Section 4.3.1, the maximum and minimum values of the effective modulus for graphene composite materials in the experimental group from the literature were calculated using the mixed formula. Subsequently, these calculated results were compared with the experimental data values reported in the literature, as illustrated in Table 8 and Figure 11.

The experimental data in the literature, as shown in Figure 11, consistently fall within the predicted range of the mixed-formula solution. This observation demonstrates that the mixed formula provides a reliable estimation range for the elastic modulus of composite materials, enabling it to guide the assessment of test data reliability and serve as a valuable tool for preliminary trend analysis in mechanical testing of composites during early stages. Consequently, this approach effectively reduces both testing costs and time requirements.

## 5. Conclusions

In this research, a formula for predicting the Young’s modulus of plate-reinforced composites is derived. Its validity is verified through comparisons with the rule of mixture (ROM), Halpin–Tsai equations, and finite-element simulations. The proposed formula incorporates the interface effect and can effectively capture the transition from the representative volume element (RVE) sandwich model to the embedded model of graphene composites. Compared with the experimental data, it is found that the experimental data fall within the modulus prediction range of the formula. This indicates that the new formula can be utilized for the preliminary trend analysis of mechanical tests of composite materials in the initial stage. As a result, it can significantly reduce the testing costs and save time.

## Figures and Tables

**Table 1:** *E*_cx_ calculated by the presented method and ROM.

*L*g = *W*g /nm	*V*g /%	Ecx/GPa		Deviation $\left(\frac{\left(1)-(2\right)}{\left(2\right)}\right)$
		Presented (1)	ROM (2)	
1	1	11.26	11.26	0.01%
	5	44.3	44.3	0.0%
	10	85.6	85.6	0.0%
5	1	12.49	12.49	0.0%
	5	50.45	50.45	0.0%
	10	97.9	97.9	0.0%
10	1	12.64	12.64	0.0%
	5	51.2	51.2	0.0%
	10	99.4	99.4	0.0%

**Table 2:** *E*_cx_ calculated by the presented method and Halpin–Tsai.

*L*g = *W*g (nm)	*V*g (%)	Ecx(GPa)		$\mathrm{Deviation}\;(\frac{\left(1)-(2\right)}{\left(2\right)})$
		**Presented** **(1)**	**Halpin–Tsai** **(2)**	
1	1	3.096	3.089	0.2%
	5	3.478	3.462	0.5%
	10	3.955	3.975	−0.5%
5	1	3.350	3.316	1.0%
	5	4.750	4.647	2.2%
	10	6.499	6.470	0.4%
10	1	3.650	3.586	1.8%
	5	6.249	6.048	3.3%
	10	9.497	9.412	0.9%

**Table 3:** Embedded represents the volume-unit model size.

Vg/%	L_m_ × W_g_ × t_m_/(nm)
1.0	3.24 × 3.24 × 3.24
3.0	2.25 × 2.25 × 2.24
5.0	1.89 × 1.89 × 1.90

**Table 4:** The sandwich RVE model prediction modulus value.

Vg/%	Non-	Linear		Exponential	
	Ecx/GPa (1)	Ecx/GPa (2)	Deviation/% $\;\;(\frac{\left(2)-(1\right)}{\left(1\right)})$	Ecx/GPa (3)	Deviation/% $\;\;(\frac{\left(3)-(1\right)}{\left(1\right)})$
1.0	11.26	16.49	46.45	14.20	26.11
3.0	27.78	43.48	56.52	36.60	31.75
5.0	44.30	70.46	59.05	59.00	33.18

**Table 5:** The embedded RVE model prediction modulus value.

Vg/%	Non-	Linear		Exponential	
	Ecx/GPa (1)	Ecx/GPa (2)	Deviation/% $\left(\frac{\left(2)-(1\right)}{\left(1\right)}\right)$	Ecx/GPa (3)	Deviation/% $\left(\frac{\left(3)-(1\right)}{\left(1\right)}\right)$
1.0	3.04	3.09	1.64	3.09	1.64
3.0	3.16	3.32	5.06	3.32	5.06
5.0	3.32	3.63	9.34	3.63	9.34

**Table 6:** The convergence of the predicted value of Ecx by the mixed formula when Lm is maximized.

Vg/%	Lg × Wg/(nm)	Lm × Wm/(nm)	Ecx/GPa
1	1 × 1	10× 10	3.033
	5 × 5	50 × 50	3.033
	10 × 10	100 × 100	3.033
	100 × 100	1000 × 1000	3.033
5	1 × 1	4.5 × 4.5	3.192
	5× 5	22.5 × 22.5	3.192
	10 × 10	45 × 45	3.192
	100 × 100	450 × 450	3.192
10	1 × 1	3.15 × 3.15	3.437
	5 × 5	10.75 × 10.75	3.438
	10 × 10	31.5 × 31.5	3.438
	100 × 100	315 × 315	3.438

**Table 7:** Data.

Test Data No.	Vg/%	Graphene Size		E_m_/GPa	E_g_/GPa
		Lg = Wg/(nm)	tg/nm		
1 [52]	0.3	1000	0.8	0.1	1000
2 [52]	0.6	1000	0.8	0.1	1000
3 [52]	1.8	1000	0.8	0.1	1000
4 [55]	0.523	1000	200	3.27	1153
5 [55]	0.523	1000	14.3	3.27	1153
6 [56]	0.6	15,000	7	2.72	1000
7 [56]	1.21	15,000	7	2.72	1000
8 [56]	1.82	15,000	7	2.72	1000
9 [56]	2.44	15,000	7	2.72	1000
10 [56]	3.06	15,000	7	2.72	1000
11 [56]	3.69	15,000	7	2.72	1000
12 [57]	05	10,000	1.7	1.0182	1000
13 [57]	1	10,000	1.7	1.0182	1000
14 [57]	2	10,000	1.7	1.0182	1000
15 [57]	4	10,000	1.7	1.0182	1000

**Table 8:** A comparison between the prediction results of the three-phase mixed formula and the experimental data.

Test Data No.	Ecx/GPa			In the Predicted Range (Y/N)
	Test Result	Formula Prediction		
		Max	Min	
1	0.2	3.13	0.1	Y
2	0.25	6.17	0.101	Y
3	1.04	18.30	0.104	Y
4	3.3	8.50	3.289	Y
5	3.65	8.46	3.289	Y
6	2.8	8.70	2.738	Y
7	2.94	14.79	2.757	Y
8	3.03	20.87	2.777	Y
9	3.11	27.05	2.798	Y
10	3.24	33.24	2.821	Y
11	3.35	39.52	2.844	Y
12	1.2111	6.013	1.024	Y
13	1.3392	11.008	1.03	Y
14	1.4800	20.998	1.042	Y
15	1.8034	40.977	1.069	Y

## Data Availability

The original contributions presented in this study are included in the article. Further inquiries can be directed to the corresponding author.
